# Tumor-associated macrophages in hepatocellular carcinoma: Cellular plasticity and therapy resistance in crosstalk

**DOI:** 10.1016/j.jpha.2025.101384

**Published:** 2025-06-30

**Authors:** Tianhao Zhang, Xi Zhao, Tingting Gao, Fang Ma

**Affiliations:** a Department of Anaesthesiology, The First Hospital of China Medical University, Shenyang, 110004, China; b Department of Anaesthesiology, Cancer Hospital of China Medical University, Cancer Hospital of Dalian University of Technology, Liaoning Cancer Hospital&Institute, Shenyang, 110042, China

**Keywords:** Hepatocellular carcinoma, Immunotherapy, Tumor microenvironment, Tumor-associated macrophages, Drug delivery

## Abstract

Hepatocellular carcinoma (HCC) is the predominant type of liver cancer. There are different risk factors for HCC including viral infection, liver fibrosis, non-alcoholic fatty liver disease, environmental factors and genomic alterations. The tumor microenvironment (TME) has been proposed as a potent regulator of tumor malignancy comprised of normal and cancerous cells. Macrophages are among the most abundant cells in the TME, known as tumor-associated macrophages (TAMs) that can control proliferation, metastasis, immune reactions and therapy response of tumor cells. In the present review, the function of TAMs in the regulation of HCC progression was evaluated. TAMs are prognostic factors in HCC that increase in TAM infiltration into TME can cause undesirable outcome in patients. Moreover, M2 polarization of macrophages can impair function of other immune cells such as T cells and natural killer (NK) cells to mediate immune evasion. TAMs demonstrate association with other biological events including autophagy and glycolysis. There is mutual interaction between TAMs and exosomes that TAM-mediated exosome secretion regulates HCC progression, while exosomes derived from other cells can also affect TAMs. Inhibition of macrophage recruitment, their depletion and increasing M1 polarization are promising approaches in HCC therapy. The natural products and nanostructures have been also recently introduced for the regulation of macrophages in HCC therapy.

## Highlights

• Tumor-associated macrophages (TAMs) significantly influence hepatocellular carcinoma (HCC) progression by promoting tumor growth, metastasis, and immune evasion within the tumor microenvironment (TME).
• The polarization of TAMs towards the M2 phenotype impairs the function of other immune cells, such as T cells and natural killer cells, facilitating immune evasion and contributing to a poor prognosis in HCC patients.
• A mutual relationship between TAMs and exosomes exists, where TAM-secreted exosomes promote HCC progression, and exosomes from other cells can modulate TAM behavior, influencing tumor dynamics.
• Targeting TAMs through inhibition of their recruitment, depletion, or promotion of M1 polarization, along with the use of natural products and nanostructures, offers promising new avenues for HCC therapy.

## Graphical abstract

**Figure:**
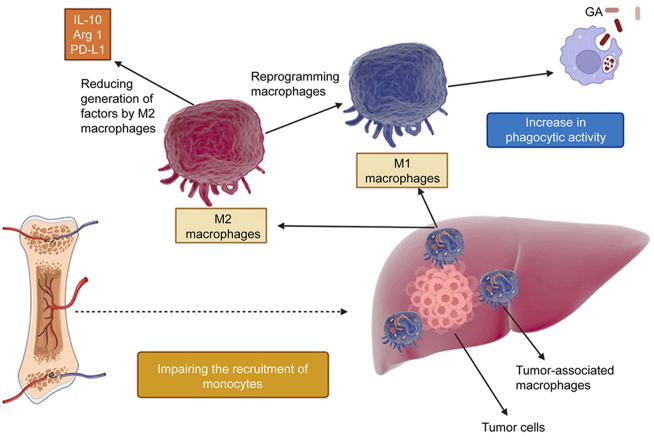

## 1 Introduction

The sixth leading causes of death in the world is liver cancer with incidence rate of 906,000 annually and from lethality point, it is the third leading cause of death with 5-year survival rate of 18% [1]. From 2000 to 2016, the incidence and lethal rates of liver cancer have increased by 43% [2,3]. Up to 75%–85% of liver cancer cases are hepatocellular carcinoma (HCC) [1]. The diseases in liver tissues including chronic hepatitis B (50% of HCC cases) or C, virus infection (hepatitis B virus (HBV) or hepatitis C virus (HCV)), alcohol consumption, and metabolic syndrome are considered as risk factors for HCC [4]. The HBV and HCV are considered as main public health problems causing one million and 450,000 deaths annually, respectively [5]. The incidence rate of HCC increases despite the advances in HBV vaccination and HCV antiviral therapies that is mainly due to the hazardous alcohol consumption and obesity [6]. In addition, inflammation and different environmental and genetic factors participate in the development of HCC [7]. Kupffer cell lineage-determining factors such as ID3 play a pivotal role in their anti-tumor activity. Specifically, ID3 allows Kupffer cells to phagocytose live tumor cells and helps recruit and activate natural killer (NK) cells and CD8 T cells, which are vital for the liver's immune surveillance and anti-tumor response. The role of ID3 in Kupffer cells has been highlighted in recent studies, showing that this factor not only enhances their phagocytic abilities but also strengthens their interactions with effector lymphoid cells, which contributes to robust anti-tumor activity in the liver [8]. This highlights the crucial role Kupffer cells play in shaping the liver cancer immune microenvironment by maintaining a balance between immune activation and suppression. Additionally, Kupffer cells interact with tumor cells through the cell-surface transmembrane protein erythroid membrane-associated protein (ERMAP), which serves as a pro-phagocytosis molecule. Tumor cells expressing ERMAP send an "eat me" signal to Kupffer cells, facilitating their engulfment and reducing the likelihood of tumor liver metastasis [9]. This mechanism further illustrates how Kupffer cells contribute to controlling tumor spread within the liver. In spite of the introduction of various therapeutic strategies including chemotherapy, surgical resection, immunotherapy and liver transplantation, among others, the treatment of HCC is still challenging. Therefore, the present review has been dedicated to evaluate the role of TAMs in the progression of HCC and then, directing the strategies for the HCC control in improving the treatment of this malignant disease.

## 2 Tumor-associated macrophages (TAMs): basics and principles

**Fig. 1:**
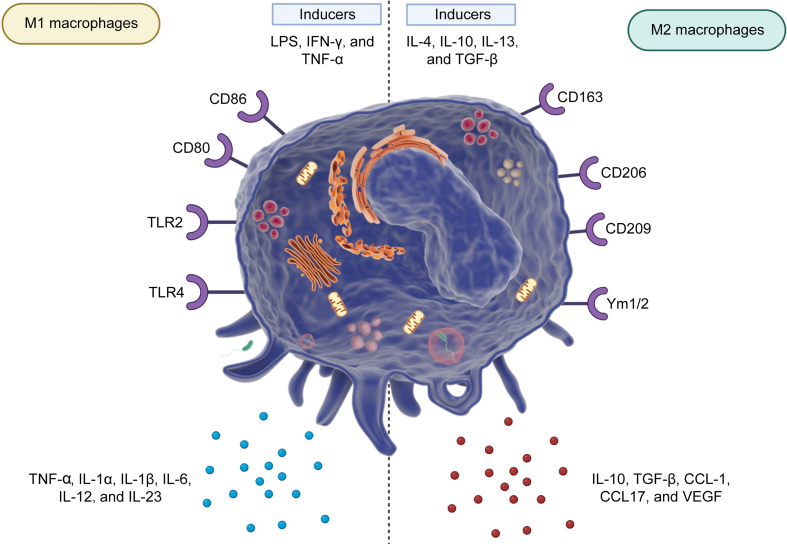
The various types of macrophages (Created by Biorender.com). There are two phenotypes including M1 and M2 polarized macrophages (Created by Biorender.com). The M1 polarized macrophages are induced by different factors including lipopolysaccharide (LPS), interferon-gamma (IFN-γ), and tumor necrosis factor alpha (TNF-α), while interleukin (IL)-4, IL-10, IL-13, and transforming growth factor-β (TGF-β) mediate M2 polarization of macrophages. There are different receptors utilized for the identification of macrophages including cluster of differentiation 163 (CD163), CD206, CD86, and CD80, among others. Moreover, they can secrete ILs and TNF to affect other cells and biological mechanism. TLR: toll-like receptor; Ym1/2: chitinase-like proteins; CCL: chemokine (C–C motif) ligand; VEGF: vascular endothelial growth factor.

The majority of TAMs result from the primitive yolk sac precursors or differentiated from the recruited bone marrow monocytes [10]. Regardless of being tissue resident or derived from bone marrow, colony-stimulating factor 1 is considered as a chemotactic factor for macrophages [11]. Moreover, the vascular endothelial growth factor A (VEGFA) can recruit and mediate differentiation of monocytes into TAMs [12]. The infiltration of blood bone marrow monocytes can differentiate into TAMs that is a response to monocyte chemoattractant protein-1 (MCP-1)/chemokine (C–C motif) ligand 2 (CCL2) generated by endothelial, stromal and cancer cells [13,14]. The TAMs present in the human body are generally originated from the monocytes of blood that a number of signalling molecules derived from cancer cells and TAMs can recruit them to change their activation and polarization [13,15,16]. Both cytokines and chemokines participate in the recruitment. The cytokines include interleukin (IL)-4, IL-13, M-CSF/colony stimulating factor 1 (CSF1), IL-10, IL-33, IL-21, and transforming growth factor-β (TGF-β), while chemokines include CCL2, CCL3, CCL15, CCL18, CX3C chemokine ligand 1 (CX3CL1), C-X-C motif chemokine ligand 8(CXCL8), and CXCL12 [17]. Notably, the macrophages can be categorized into two phenotypes according to their function. The M1 macrophages can be induced by the inflammatory factors including bacterial lipopolysaccharide (LPS) and interferon-gamma (IFN-γ) contributing to the Th1-induced immune reactions, mediating antibacterial protection and antigen presentation using major histocompatibility complex-II (MHC-II). In order to support the tissues against bacteria, the M1 polarized macrophages generate inflammatory cytokines including tumor necrosis factor alpha (TNF-α), IL-1α, IL-1β, IL-6, IL-12, and IL-23 to enhance inducible nitric oxide synthase (iNOS) levels and promote nitric oxide (NO) in the extracellular matrix in enhancing reactive oxygen species (ROS) levels. The biomarkers for the M1 polarized macrophages are human leukocyte antigen-DR isotype (HLA-DR), CD80, CD86, and MHC-II [18]. On the other hand, there are M2 polarized macrophages that can be induced by cytokines including IL-4, IL-10, IL-13, and TGF-β. The M2 macrophages exert anti-inflammatory function. Moreover, M2 macrophages stimulate Th2-induced humoral responses against helmints. The M2 macrophages can improve tissue healing and reconstruction of bone tissue [19]. Fig. 1 shows the different kinds of macrophages.

## 3 Origin and function of liver macrophages

There is a significant heterogeneity in the intrahepatic macrophage pools [20]. There are two kinds of liver macrophages relying on their origin and activity. There is origination of Kupffer cells from erythromyeloid progenitor cells in the fetal yolk sac. Kupffer cells are resident in the liver tissue and they are nonmigratory macrophages with the capacity of self-regeneration [21]. These cells are observed in the hepatic sinusoids that are different from circulating monocyte-derived macrophages originating from bone marrow [22]. Kupffer cells and monocyte-derived macrophages have a number of similar phenotypes and it is difficult to provide their differentiation because of lacking lineage-specific markers [22]. Several monocyte-derived macrophages can differentiate and demonstrate a phenotype that can be similar to Kupffer cells in certain situations [23,24]. C-type lectin domain family 4 member F (Clec4F) and T cell immunoglobulin and mucin-domain containing 4 (Tim4) have been utilized as markers for monocyte-derived macrophages from Kupffer cells [25].

## 4 Liver macrophages in the HCC pathogenesis and progression

One of the features of solid tumors is the infiltration of macrophages [25]. HCC is considered as a factor involved in the inflammation-associated cancer. The different inflammation-associated risk factors can lead to the chronic inflammation in the liver tissue. On the other hand, chronic inflammation results in cycles of destruction-regeneration in the liver, resulting in the fibrosis and cirrhosis, finally causing HCC pathogenesis. The liver macrophages are considered as the modulators of this event [26,27]. There is high infiltration of TAMs in HCC with M2 polarization. A common feature of liver TAMs is CD68 and in order to differentiate between M1 and M2 macrophages, CD86 is used as a M1 marker, while CD163 and CD206 are utilized as M2 markers [28]. The malignant characteristic of HCC can be achieved through low levels of CD86^+^ M1 macrophages and increased levels of CD206+ M2 macrophages, highlighting the function of CD86 and CD206 as prognostic factors [29]. Multiple factors participate in the recruitment and migration of monocyte-derived macrophages provided by chemokines such as CCL2, CCL5, CCL15, CCL20, and cytokines including CSF-1 47–49 [30].

## 5 TAMs and related pathways as prognostic factors in HCC

In the recent years, the macrophages and related pathways have been considered as the reliable prognostic factors in HCC. Since HCC is a malignant disease, the macrophages can predict the responses of tumor cells to therapy and also, affect the overall survival and outcome in patients. The macrophage-related factors have been considered as prognostic factors in HCC and among them cytotoxic T-lymphocyte associated protein 4 (CTLA4) and TNF receptor superfamily member 9 (TNFRSF9) demonstrate overexpression in high-risk patients, regulating response of patients to immunotherapy [31]. The single-cell sequencing studies have been beneficial in understanding the function of macrophages in HCC. Notably, both cancer stem cells and secreted phosphoprotein 1 (SPP1)+ macrophages display co-localization in the hypoxic region, providing unfavourable prognosis [32]. Another experiment based on single-cell sequencing highlights the CK19+ cancer stem cells and SPP1+ TAMs niche in HBV-associated HCC [33]. The multi-omics analysis has been also beneficial in understanding macrophage-related receptor and ligand as prognostic factor in HCC and among them, SPP1, angiopoietin-2 (ANGPT2), and nucleolin (NCL) are critical biological factors in HCC [34]. Table S1 is a summary of TAMs in HCC progression and was provided in the Supplementary data.

## 6 Polarization and infiltration of TAMs in HCC

**Fig. 2:**
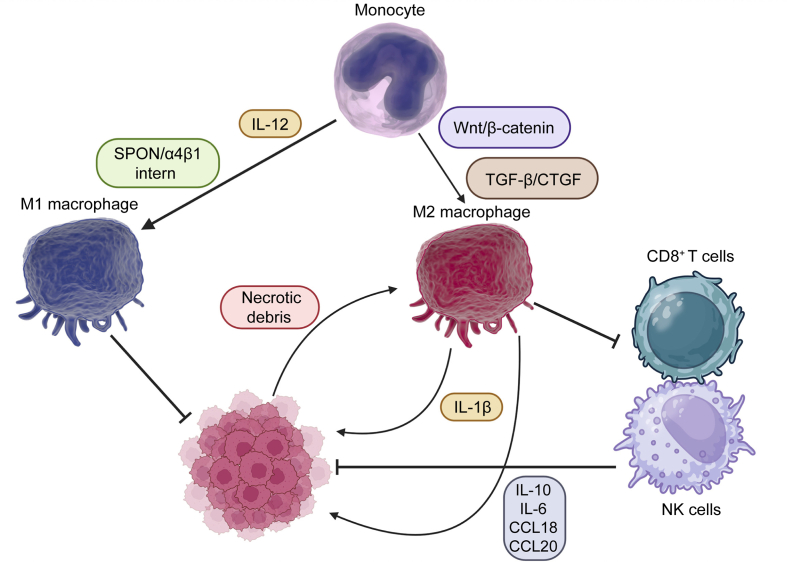
The polarization of macrophages in hepatocellular carcinoma (HCC) (Created by Bioender.com). Monocytes can be transformed to M1 macrophages by stimuli including interleukin (IL)-12 and spondin-1/integrin β1 (SPON/α4β1) intern. On the other hand, monocytes can be transformed to M2 macrophages by Wnt and transforming growth factor β (TGF-β). Moreover, necrotic debris from tumor cells can enhance M2 polarization of macrophages. The generation of M1 macrophages can suppress progression of HCC. The M2 polarized macrophages can suppress the collaborative function of natural killer (NK) cells and CD8^+^ T cells. The M2 macrophages enhance progression of HCC through generation of IL-1β, IL-10, IL-6, chemokine (C–C motif) ligand 18 (CCL18), and CCL20 [44]. Wnt/β-catenin: wingless-related integration site/beta-catenin; CTGF: connective tissue growth factor; CDP-T cells: common dendritic progenitor T cells.

The function of TAMs in the regulation of HCC progression is obvious. As a result, the increasing evidences have focused on understanding the molecular pathways regulating TAM polarization and infiltration in HCC. Hsa_circ_0010882 is a regulator of TAM polarization in HCC. Notably, circ-0010882 shows upregulation in HCC, association with unfavourable prognosis. Silencing circ-0010882 enhances the M1 polarization of macrophages (approved by presence of markers including TNF-α and iNOS), whereas it diminishes M2 polarization of macrophages (approved by the presence of markers including Arg-1 and CD206). Such enhancement in M1 polarization impairs the migration and invasion of HCC cells [35]. On the other hand, the upregulation of wingless-related integration site/beta-catenin (Wnt/β-catenin) by LINC00662 can stimulate M2 polarization of macrophages to HCC malignancy [36]. As a result, increasing the recruitment of M1 macrophages can impair HCC. In this regard, SPON2-α4β1 integrin can induce RhoA and Rac1 and enhance F-actin organization along with enhancing infiltration of M1 macrophages. The enhancement in F-actin accumulation can upregulate Hippo through reducing LATS1 phosphorylation to enhance nuclear transfer of YAP. These events are vital for impairing HCC [37]. Moreover, there are factors capable of reducing M2 polarization of macrophages in HCC therapy. The proprotein convertase subtilisin/kexin type 9 (PCSK9) has been shown to upregulate OX40L to induce M1 polarization of macrophages and suppress HCC proliferation [38]. These studies highlight that increase in M2 polarization can enhance invasion of HCC cells. The reason is based on that matrix metalloproteinase (MMP)-9 and MMP-2 can be derived from macrophages to mediate rupture of fibrous capsule of HCC in enhancing metastasis of cancer cells [39]. However, the results about the function of macrophages in HCC progression are controversial. An example is the study performed by Kazankov et al. [40] showing that CD163 as a macrophage activation marker does not have association with size and number of HCC as well as metastasis and invasion. As a result, both polarization and migration control of macrophages can reduce HCC progression. For the recruitment of macrophages, it is vital to secrete a number of chemokines and cytokines. High-mobility group AT-Hook 1 (HMGA1) has been suggested to increase CCL2 levels through nuclear factor kappa B (NF-κB) to increase macrophage recruitment in HCC [41]. In addition to recruitment and polarization, the differentiation of macrophages can be controlled in regulating HCC malignancy. The extosomal pyruvate kinase M2 (PKM2) can be derived from HCC cells that along with metabolic reprogramming in monocytes, it can cause signal transducer and activator of transcription 3 (STAT3) phosphorylation to enhance levels of factors in promoting differentiation of monocytes into macrophages, mediating tumor microenvironment (TME) remodelling [42]. When the differentiation of monocytes into macrophages occurs, the prostaglandin E2 (PGE2) is derived from macrophages that can interact with the tumor UHRF1 in enhancing progression of HCC cells [43]. These experiments demonstrate that multiple factors participate in the regulation of polarization, recruitment and differentiation of TAMs in the TME of HCC (Fig. 2).

## 7 Hepatic stellate cells (HSCs) and macrophages in HCC

There are a number of different cell types that make up the hepatic microenvironment, which includes immune cells, hepatocytes, and HSCs [45]. These cell types all have a role in the development of HCC. It is important to highlight that activated HSCs cause phenotypic alterations and boost cell proliferation. On the other hand, HSCs induce the production of cytokines, chemokines, MMPs, and growth factors, which may exacerbate hepatic inflammation and accelerate the development of HCC by inducing EMT [[46], [47], [48], [49]]. Activated HSCs, on the other hand, are responsible for the production of chemokines, which are used to recruit immune cells. These immune cells then proceed to activate HSCs by the secretion of cytokines or through direct crosstalk between cells [[50], [51], [52]]. J5 cells, which are intermediately differentiated human liver cancer cells, were co-cultured with HSC-conditioned medium (HSC-CM) [53]. The changes in cell phenotypic and cytokine profiles were studied in order to determine the influence that HSCs have on the development of hepatoma. Therefore, the administration of CM secreted by activated HSC (aHSC-CM) could induce the expression of N-cadherin, cell migration, and invasive potential in J5 cells, as well as the activity of MMPs, which suggests that aHSC-CM could trigger the EMT. The stage of liver fibrosis was found to have a significant correlation with the grade of the tumor. After that, the HSC-CM was investigated further, and network analysis revealed that particular cytokines and soluble proteins, such as activin A, that are released from activated HSCs have the potential to significantly influence the tumor-associated immune microenvironment that is involved in macrophage polarization. This, in turn, would reduce the immune surveillance of a host and cause hepatoma cells to exhibit a more malignant phenotype. It was shown that a subpopulation of macrophages exclusively expressed Arg1 and Cx3cr1 in the peritumoral region of HCC patients, and that these macrophages were substantially enriched with genes associated to retinol metabolism [54]. This was discovered by single cell RNA-sequencing research. In the area proximal to the HCC, when activated HSCs (aHSCs) expressing α-SMA displayed co-localized expression of CX3CL1, flow cytometry analysis revealed a substantial increase in the frequencies of CD14+CD11b + HLA-DR‒ macrophages when they were paired with CX3CR1. As a result, the expression of CX3CL1 messenger RNA (mRNA) was significantly elevated in aHSCs inside the surrounding HCC in mice that were carrying tumors. This was accompanied by an infiltration of CX3CR1+Ly6C + macrophages, which was mostly observed in conjunction with a reduction in CD8^+^ T cells. We demonstrated that CX3CR1+Ly6C + macrophages moved and strongly expressed arginase-1 by connecting with retinoid-enriched aHSCs in the neighboring HCC by adoptive transfer and *in vitro* co-culture of myeloid cells. This was accomplished through the development of myeloid cells. Arginase-1 expression was considerably enhanced in CX3CR1+Ly6C + macrophages and human blood CD14^+^ cells as a result of direct treatment with retinoids or co-culturing with retinol-storing mouse aHSCs or human LX-2 cells. This resulted in the reduction of CD8^+^ T cell proliferation. Furthermore, the development of HCC was significantly slowed down by either a genetic deficit of CX3CR1 in myeloid cells or a pharmacological suppression of retinol metabolism.

## 8 EMT and macrophages in HCC

The interaction of macrophages and tumor cells can change EMT in HCC. Oxoaporphine Pr(III) complex enhances the levels of CD86, TNF-α, and IL-1β, whereas it decreases levels of CD163, CD206, CCL2, and VEGFA in macrophages. Moreover, this complex has been able to downregulate AMP-activated protein kinase (AMPK) and induce nuclear factor kappa B (NF-κB) by enhancing RelA/NF-κB p65 subunit (p65) Ser536 phosphorylation to suppress M2 polarization of macrophages. Such changes in molecular pathways can reduce levels of Snail transcription factor (Snail) and CCL2 to suppress EMT in HCC [55]. The long non-coding RNA Ma301 (lnc-Ma301) has been suggested as a macrophage polarization-related factor with poor expression in HCC. The upregulation of lnc-Ma301 and its interaction with caprin-1 can suppress EMT and mediate M1 polarization of macrophages in impairing metastasis of tumor cells [56]. However, this experiment does not provide any direct interaction between macrophages and EMT that requires further validation. The IL-35 is able to mediate EMT in HCC. IL-35 upregulates STAT3 and promotes the M2 polarization of macrophages to stimulate EMT in enhancing metastasis of HCC cells [57]. This provides a new insight that cytokines and pro-inflammatory cytokines are critical regulators of macrophage polarization and EMT in HCC. The interesting point is the capacity of macrophages in the secretion of cytokines. Notably, IL-8 can be secreted by macrophages that induces Janus kinase 2 (JAK2)/STAT3/Snail axis, causing EMT and increasing metastasis of HCC cells [58]. Co-culture of tumor cells with M2 polarized macrophages can induce EMT, further highlighting the interaction of macrophages and EMT [59].

## 9 Autophagy and macrophages in HCC

In the recent years, the interaction of autophagy and macrophage in case of HCC has been of importance. The loss of autophagy in macrophages can increase microvascular metastasis and cause unfavourable prognosis. HCC is able to increase mammalian target of rapamycin (mTOR) levels in impairing autophagy in macrophages. The autophagy inhibition through silencing ATGs can further elevate the invasion of HCC cells. The autophagy deficiency increases the levels and accumulation of NLRP3 inflammasome to enhance the release and cleavage of IL-1β, accelerating HCC progression and causing metastasis through EMT induction. The loss of autophagy also increases the recruitment of macrophages through CCL20/C–C motif chemokine receptor 6 (CCR6) axis [60]. In this case, the application of autophagy inducers can mediate autophagy in macrophage to prevent their recruitment and impair EMT-mediated metastasis in HCC. The anti-cancer activity of autophagy can also change the polarization of macrophages. The reduction of autophagy can enhance the M2 polarization of macrophages that is related to the enhanced instability of NF-κB in HCC. Upon autophagy loss, the TAB3 instability enhances through ubiquitination that inhibits NF-κB, increasing M2 polarization of macrophages in HCC [61]. The lack of autophagy in macrophages can increase the expression level of factors associated with immunosuppression [62]. Therefore, the pharmacological regulation of autophagy can improve the chance in cancer therapy. Quercetin has been exploited to suppress HCC through autophagy induction and enhancing M1 polarization of macrophages [63].

## 10 Glycolysis, lipid metabolism and macrophages in HCC

**Fig. 3:**
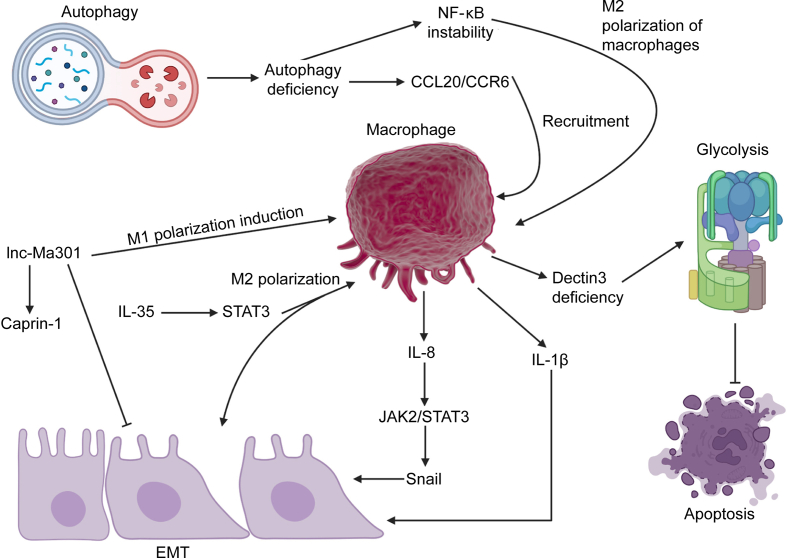
The function of epithelial-mesenchymal transition (EMT), autophagy and glycolysis in interaction with macrophages (Created by Biorender.com). The loss of autophagy increases nuclear factor kappa B (NF-κB) instability and induces chemokine (C–C motif) ligand 20 (CCL20)/C–C motif chemokine receptor 6 (CCR6) axis to enhance recruitment and M2 polarization of macrophages. Moreover, Dectin3 deficiency in macrophages can induce glycolysis to impair apoptosis in tumor cells. The macrophages can secrete interleukin (IL)-8 and IL-1β to mediate EMT in cancer metastasis via affecting various molecular pathways such as Janus kinase 2 (JAK2)/signal transducer and activator of transcription 3 (STAT3). Moreover, IL-35 upregulates STAT3 to increase M2 polarization of macrophages in EMT induction. lnc-Ma301: long non-coding RNA Ma301; Snail: Snail transcription factor.

In the recent years, the interaction of macrophages and glycolysis has been of importance in the regulation of HCC progression. The loss of Dectin3 in mice can enhance tumor volume and promote the levels of macrophages along with a reduction in CD4^+^ and CD8^+^ cells. Dectin3 efficiency in macrophages induces glycolysis to enhance HCC proliferation and reduce apoptosis [64]. The HCC cells are able to secrete IL-10 and TGF-β to induce M2 polarization of macrophages. In M2 macrophages, there is upregulation of Wnt2b to mediate β-catenin, undergoing nuclear translocation to increase c-Myc levels in glycolysis induction. Then, M2 polarized TAMs induce EMT in HCC metastasis [65]. In some cases, long noncoding RNA (lncRNA) miR4458HG can affect both TAMs and glycolysis [66], but this experiment did not provide an interaction of TAMs and glycolysis in HCC. However, the idea is that glycolysis induction enhances M2 polarization of macrophages. The upregulation of epithelial cell transforming 2 (ECT2) enhances polo-like kinase 1 (PLK1) to downregulate phosphatase and tensin homolog (PTEN) for induction of glycolysis, increasing M2 polarization to impair NK and T cells [67]. The induction of glutaminolysis that changes glutamine into glutamate to generate α-KG. In this case, aspartate is entered into urea cycle to produce spermidine. Then, spermidine generates eIF5A-hypusineto mediate translation of hypoxia-inducible factor 1 alpha (HIF-1α), causing glycolysis to induce M2 polarization of macrophages [68]. Fig. 3 reveals the function of EMT, glycolysis and autophagy in interaction with macrophages in HCC.

In addition to glycolysis, the recent studies have highlighted the dysregulation of lipid metabolism in HCC [[69], [70], [71], [72]]. Notably, changes in lipid metabolism in TAMs can affect HCC progression. A number of processes are included in the realm of lipid transport, including absorption, excretion, transport, and utilization activities. Through its participation in signal regulation and other tasks, it plays an important part in the process of providing synthetic metabolic precursors for HCC and TAMs with the necessary precursors [73]. In general, the raw materials for lipid metabolism are acquired from both external intake and de novo synthesis. Among the several forms of lipids that are exogenously ingested and employed for energy generation, FA is the most important type. It has been demonstrated that macrophages are capable of endocytosing a variety of low-density lipoproteins, including acLDL and ox-LDL, through processes that are mediated by scavenger receptors (SR) [74]. TAMs have been shown to promote FA absorption inside the TME by upregulating the expression of the FA translocase CD36, which is a sort of scavenger receptor [75]. This has been established through animal experimental proof. In addition, the sequencing of RNA from a single cell in phase III human HCC samples has shown that TAMs had an increased expression of FA-binding protein 1 (FABP1). This overexpression, in combination with the peroxisome proliferator-activated receptor γ (PPARG)/CD36, has a role in facilitating the transport and oxidation of fatty acids [76]. The synthesis of TG with glycerol is the usual method by which TAMs store excess FA intracellularly in the form of lipid droplets (LD). As a consequence of this, TAMs, in conjunction with HCC cells, increase the expression of FABP in response to FA stimulation. This, in turn, regulates the absorption and storage of lipids within the microenvironment of high-fat HCC [77]. On the other hand, new research has brought to light the fact that lipid accumulation is an essential characteristic of HCC, with high levels of LD being regarded suggestive of malignant invasion across a variety of tumor forms [78]. Therefore, we hypothesize that the accumulation of lipids in TAMs may fulfill the role of a nutritional reserve for the advancement of HCC and may also play a role in the promotion of tumors. Reprogramming of lipid metabolism in TAMs is an essential component for the polarization of macrophages and the development of hepatocarcinogenesis [79]. Through the activation of PPAR, the downregulation of receptor-interacting protein kinase 3 (RIPK3) in TAMs led to a reduction in ROS levels and a promotion of fatty acid metabolism, which contributed to the accumulation and polarization of M2 TAMs [80]. Fatty acid oxidation (FAO) blocking is a promising method for decreasing tumor growth in HCC. It is also capable of reversing the immunosuppressive function of TAMs.

## 11 TAMs in the regulation of immune system in HCC

**Fig. 4:**
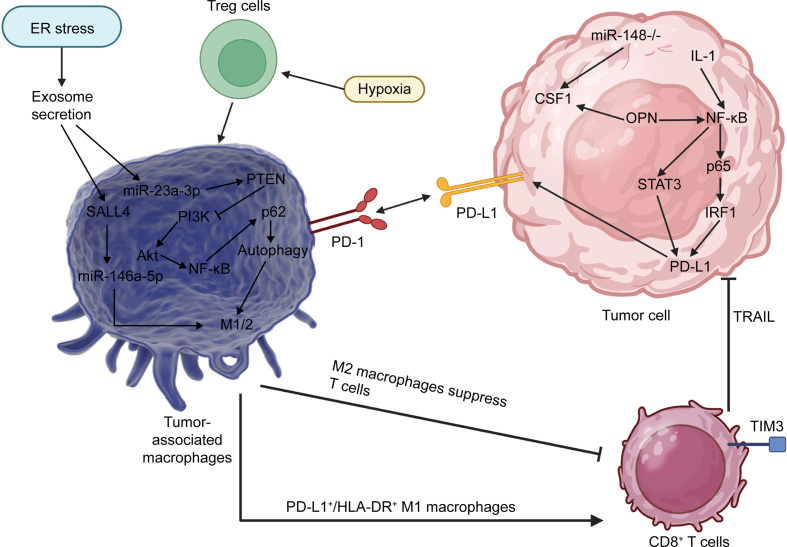
The tumor-associated macrophages (TAMs) as key players in the modulation of hepatocellular carcinoma (HCC) immunity (Created by Biorender.com). The presence of endoplasmic reticulum (ER) stress can cause exosome secretion to make changes in macrophages including Sal-like protein 4 (SALL4) upregulation in enhancing microRNA-146a-5p (miR-146a-5p) levels to change polarization of macrophages. The hypoxia enhances infiltration and population of Treg cells to induce M2 polarization of macrophages. The nuclear factor kappa B (NF-κB) p62 subunit (p62) upregulation by NF-κB promotes autophagy to induce M1 polarization. M2 macrophages suppress the function of CD8^+^ T cells, while M1 macrophages enhance activity of T cells in cancer immunotherapy. The role of interferon regulatory factor 1 (IRF1) and signal transducer and activator of transcription 3 (STAT3) in increasing programmed death-ligand 1 (PD-L1) expression can mediate immune evasion [86]. P18K: Phosphoinositide 3-kinase, AKI: Acute Kidney Injury, M1/2: M1/M2 Macrophages, Treg cells: Regulatory T cells, PD-1: Programmed cell death protein 1, PD-L17/HLA-DR M1 macrophages∗: Programmed death-ligand 1 and HLA-DR positive M1 macrophages, TNM3: Tumor Node Metastasis staging system, CSF1: colony stimulating factor 1, IL-1: Interleukin 1, TPD-L1: Tumor-associated PD-L1, TRALL: Tumor necrosis factor-related apoptosis-inducing ligand, M2 macrophages suppress: M2 macrophages suppression.

In the recent years, the cancer immunotherapy has been emerged as an ideal candidate in the tumor suppression. The immunotherapy uses the host's immune system to combat against cancer. The tumor-associated antigens are identified by the immune cells and finally activate T cells. However, the tumor cells have been able to develop resistance into immunotherapy, known as immune evasion [81]. The interactions occurring in the TME can regulate immune responses. An example is the function of CAFs in providing immunosuppressive TME [82]. In addition, the TAMs in the TME can regulate the immune responses. The polarization of macrophages into M2 can impair the immune responses. The overexpression of SLFN11 occurs in cancer cells. Loss of SLFN11 can promote the levels of macrophages in TME and enhance tumorigenesis. The loss of SLFN11 can enhance the migration of macrophages and promote M2 polarization of macrophages using CCL2. Noteworthy, SLFN11 can suppress Notch and CCL2 through competitive binding to tripartite motif containing 21, impairing TRM21-induced RBM10 degradation. This increases the stability of RBM10 to upregulate NUMB exon 9 skipping. The application of CCR2 antagonist can enhance the impact of anti-programmed cell death protein 1 (anti-PD-1) in cancer therapy [83]. A number of macrophages are programmed death-ligand 1 (PD-L1) positive that their infiltration into TME can increase tumorigenesis. There is upregulation of ALKBH5 in HCC and it can mediate unfavourable prognosis. The function of ALKBH5 in HCC demonstrated that it can accelerate cancer growth and invasion as well recruits the PD-L1 positive macrophages. ALKBH5 enhances the levels of c-Jun N-terminal kinase (JNK) and extracellular signal-regulated kinase (ERK) through promoting MAP3K8 levels, affecting IL-8 levels and increasing the recruitment of macrophages [84]. Since the M2 polarization of macrophages can increase the tumorigenesis and trigger an immunosuppressive TME, the genes related to M2 polarization of macrophages have been identified including *PDLIM3*, *PAM*, *PDLIM7*, *FSCN1*, *DPYSL2*, *ARID5B*, *LGALS3*, and *KLF2* [85]. Therefore, such factors can be targeted for impairing the M2 polarization of macrophages, reducing immunosuppressive TME and improving prognosis and survival of HCC patients. Hence, it can be concluded that TAMs can provide an immunosuppressive TME and this can impair the process of carcinogenesis. Moreover, TAMs can cause T cell exhaustion, while they increase levels of Treg cells in mediating immune evasion. The suppression of M2 polarized macrophages can increase the potential of cancer immunotherapy, especially PD-L1 blockade in HCC elimination. Fig. 4 highlights the function of TAMs in the regulation of immune system in HCC cells.

## 12 Angiogenesis and macrophages in HCC

The accumulation of TAMs has been shown to occur in poorly vascularized necrotic regions [87]. Such regions have been shown to become hypoxic and this can result in pro-angiogenesis of TAMs [88]. The increasing evidences have shown that there is association between high density of TAMs and microvessel density. TAM count has been shown to increase microvessel density in HCC [89]. The TAMs demonstrate expression of various angiogenic factors in human cancers such as vascular endothelial growth factor (VEGF) [90] and thymidine phosphorylase [91]. The TAM-derived proteases can induce angiogenesis, since extracellular proteolysis is essential for the generation of new vessels. The macrophages have capacity of secreting proteinases to release a number of angiogenic factors binding to the heparin sulfate in proteoglycans and fragment fibrin and collagen stimulating angiogenesis. MMP-1, -2, -3, -9 and -12, plasmin, uPA and its receptor are proteinases capable of angiogenesis induction. The TAMs have been shown to be main source of MMP-9 and synthesize uPA in the different human cancers [92,93].

## 13 TAMs and exosomes interaction in HCC

**Fig. 5:**
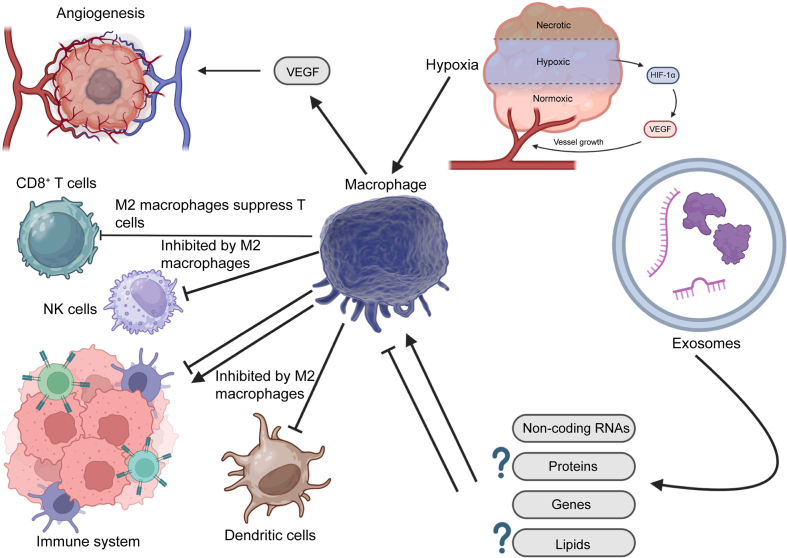
The regulation of angiogenesis, exosomes and immune reactions by macrophages (Created by Biorender.com). The presence of hypoxia increases levels of hypoxia-inducible factor 1 alpha (HIF-1α) affecting macrophages to secrete VEGF in angiogenesis induction. Moreover, M2 macrophages impair function of immune cells including dendritic cells, natural killer (NK) cells and CD8^+^ T cells. Exosomes can transfer genes and non-coding RNAs to regulate macrophage polarization, while more focus should be directed towards transfer of proteins and lipids by exosomes in macrophage polarization regulation. GIFD: Growth and Differentiation Factor.

As extracellular vesicles, exosomes have a particle size of 30–150 nm that their secretion occurs upon the fusion of multivesicular bodies with the plasma membrane [38]. After the discovery of exosomes in 1980s [94,95], the science and knowledge towards exosomes have been improved. The various kind of therapeutics including proteins, lipids and nucleic acids can be enriched in exosomes [96]. The exosomes are beneficial for preserving homeostasis and stress control by the transfer of functional bioactive molecules among the cells. The exosomes participate in the regulation of tumor-associated processes such as apoptosis, metastasis, angiogenesis, immune evasion and drug resistance. These roles have been investigated from the various viewpoints including donor cells or content type [[97], [98], [99], [100], [101]]. Many of the cells have the capacity of secreting exosomes. These cells utilize exosomes as a manner in changing the target cells and in case of cancer, both tumor-suppressing and –promoting functions of exosomes have been highlighted. Although cancer cells have higher ability in exosome secretion compared to the normal cells, exosomes can be secreted by other cells, especially those in the TME. TAMs and exosomes demonstrate mutual interaction in the regulation of HCC progression. A potential way can be considered the regulation of metabolic reprogramming in HCC cells. The TAMs have shown potential in the secretion of exosomes. The TAM-derived exosomes can transfer lncMMPA to downregulate miR-548s. This promotes the expression of ALDH1A3 to induce glycolysis and increase the glucose metabolism in HCC [102]. Inhibiting the biogenesis of exosomes can impair the transfer of this lncRNA, further suppressing glycolysis in HCC elimination. On the other hand, the exosome can transfer bioactive molecules into TAMs in regulating HCC progression. The CD8^+^ T cell inhibition can occur in HCC. In this way, GOLM1 reduces the ubiquitination of PD-L1 by COP9 signalosome 5. This enhances the transfer of PD-L1 by exosomes through downregulating Rab27b, transferring to TAMs to impair the function of CD8^+^ T cells [103]. Fig. 5 highlights the angiogenesis regulation, exosome release and immune reactions by macrophages in HCC.

## 14 Therapeutic regulation of TAMs in HCC

One of the strategies for the regulation of macrophages in HCC therapy is based on impairing the recruitment of monocytes/macrophages [104]. The suppression of CCL2/CCR2 axis with the application of CCR2 antagonist impairs cancer progression and improves the potential of sorafenib in HCC therapy [105,106]. Another strategy to diminish the recruitment of macrophages is based on suppressing CSF1/CSFR1 axis capable of affecting differentiation and survival factors [107,108]. CSF1 can be derived from HCC cells that its transcriptional regulation is performed by ZFP64, mediating M2 polarization of macrophages and causing anti-PD-1 resistance [109]. PKCα/ZFP64/CSF1 axis downregulation using lenvatinib has been shown reduce PKC expression [110] to accelerate the potential of anti-PD-1 therapy [109,111]. The application of PLX3397 as the inhibitor of colony-stimulating factor 1 receptor (CSF1R) can also suppress HCC progression through re-education of TAMs [112]. The downregulation of CSF1/CSFR1 can decrease the recruitment of macrophages and suppress M2 polarization of macrophages, enhancing sensitivity of HCC cells to anti-PD-1 therapy [113]. Generally, the depletion of macrophages has been considered as an ideal strategy for HCC therapy [114]. The transient inhibition of macrophages has been also beneficial in diminishing the TAM-induced immunosuppression [115]. Zoledronate acid (ZA) has been applied as a drug that its combination with sorafenib can significantly reduce the infiltration of macrophages and impair the metastasis in liver cancer model [116]. Combretastatin A-1 phosphate is a kind of microtubule polymerization inhibition capable of downregulating Wnt to trigger apoptosis in TAMs [117]. However, there are still a number of difficulties regarding the depletion of macrophages. There is an imbalance of the concentration distribution at the various sites [115]. Moreover, the selective depletion of macrophages should be performed to specifically affect M2 polarized macrophages.

For the purpose of enhancing immune cells' ability to recognize cancer, the chimeric antigen receptor (CAR), has emerged as a potentially useful strategy [118]. TAMs that have been generated with CAR designs have demonstrated adequate potency, as evidenced by their capacity to infiltrate solid tumors and their ability to travel through the inhibitory transfer medium. As is the case with CAR-T, the core components of CAR-M consist of an extracellular domain that enables specific recognition by a single-chain variable fragment (scFv) (for example, CD19 and HER2), a hinge domain, a transmembrane domain (primarily CD8), and an intracellular domain that demonstrates dedicated downstream signaling (for example, CD3ν and FcγR). CAR-M cells of the first generation are changed with altered CARs in order to target particular antigens in order to recognize tumor cells and boost their ability to phagocytose. The CAR-M structure, on the other hand, only makes advantage of the distinctive characteristics of macrophages, primarily phagocytosis. According to Morrissey et al. [119], CAR-phagocytes (CAR-Ps) were built in order to improve phagocytosis. This structure serves as a sample example. The ability of macrophages to penetrate other cells was utilized to its fullest extent in the first generation. Activating the production of MMPs, which are induced by CD147 and have the ability to destroy the extracellular matrix of the tumor in order to overcome physical barriers, was accomplished by Zhang et al. [120] by the construction of CAR-HER2-CD147. These statements provide the fact that CAR macrophages can provide new insights in the treatment of cancer. However, the data regarding HCC is poor and more studies should be conducted in terms of improving phagocytic activity of macrophages. It has been demonstrated that GPC3-targeted CAR T cell treatment has a high safety profile in patients who have been diagnosed with HCC in a phase I clinical study [114,121]. mCAR-T cells may be stimulated to create immune-activated IFN-γ by the promotion of IL-12 production from TAMs, which in turn enhances the anti-HCC action of GPC3-mCAR T cells [122].

The immune system plays a crucial role in tumor suppression, with macrophages being key players in both innate and adaptive immune responses, and in regulating inflammation [123]. Various anti-inflammatory factors such as TGF-β1, IL-10, IL-4, and PGE2 can induce M2 polarization of macrophages [124], which are the predominant type in tumors. M2 macrophages are characterized by low expression of certain antigens like CD51 and carboxypeptidase M, and high expression of IL-10, IL-6, among others, while also secreting chemokines like CCL17 and CCL22 [[125], [126], [127]]. The deletion of GSK3β in macrophages reduces tumorigenesis and enhances sensitivity to PD-1 blockade [128], while inhibiting CacyBP prevents macrophage recruitment and improves PD-1 blockade function in immunotherapy [129]. TAMs mediate immunosuppression in HCC, but CKI can counteract this by boosting pro-inflammatory responses and enhancing CD8^+^ T cell function [130]. Bufalin promotes M1 polarization, boosting anti-cancer immunity [131]. M2 macrophages also secrete extracellular vesicles that induce immune evasion by transferring IQGAP1 to the nucleus and enhancing STAT3 phosphorylation [132]. Conversely, inducing M2 polarization through COX-2 expression leads to T cell exhaustion [133]. There are efforts to regulate macrophage polarization in HCC immunotherapy, including a Listeria-based vaccine that promotes M1 polarization through NF-κB activation, potentially sensitizing HCC cells to anti-PD-1 therapy [134].

## 15 Nanoparticles and macrophages in HCC

Regarding the advances in field of nanobiotechnology, the experiments have focused on the application of nanoparticles for TME remodelling that macrophage regulation is among them. The nanostructures can increase macrophage depletion. The liposomal nanoparticles have been developed based on thin film hydration strategy and application of ammonium sulfate gradient to co-deliver doxorubicin and clondronate. These nanoparticles had particle size of 180–200 nm and can suppress relapse and progression of liver tumor. Moreover, they can decrease the number of CD68^+^ macrophages and causing TME remodelling [135]. The inflammation caused by the macrophages can also increase the hepatocarcinogenesis. Therefore, one of the ideas is related to the modulation of inflammatory pathways. Notably, the liposomes have been embedded with 3,5,3′-triiodothyronine to suppress the secretion of pro-inflammatory factors by macrophages in preventing hepatocarcinogenesis [136]. The nanostructures can also re-educate the macrophage for improving the response to chemotherapy. In this way, pH-sensitive nanoparticles have been developed to co-deliver sorafenib and modified resiquimod. The development of polymeric nanoparticles was based on application of methoxyl-poly(ethylene glycol)-b-poly(lactic-co-glycolic acid), possessing pH-sensitive feature. The intravenous injection of these nanostructures increases their accumulation at the tumor site and respond to the pH of TME, leading to the separation of surface polyethylene glycol (PEG) chains. Such PEG separation is essential for increasing uptake by TAMs and HCCs, increasing M1 polarization of macrophages and promoting sorafenib sensitivity in HCC cells [137]. Although the main focus of the studies has been on the application of bioengineered nanoparticles in the laboratory, the exosomes and extracellular vesicles are also beneficial in the delivery that was discussed in previous sections. However, since the extracellular vesicles can regulate TAM polarization in HCC [138], their modification for improving their selectivity and specificity can improve the cancer therapy. Table S2 summarizes the potential of nanoparticles for the regulation of TAMs in HCC therapy, provided in the Supplementary data.

## 16 Conclusion and future perspectives

The most common type of liver cancer is HCC. In spite of the development of different therapeutics for HCC, the treatment of this disease is challenging because of drug resistance and tumor recurrence. As a result, the studies have focused on understanding the underlying factors involved in HCC progression to develop novel therapeutics. The TME remodelling commonly occurs in HCC and it is responsible for the increase in the tumorigenesis and a number of aggressive behaviour of cancer cells including proliferation and metastasis. Macrophages are among the most abundant cells in the TME and this is also true for the HCC cells. Significant efforts have been made in understanding the function of macrophages in the regulation of HCC progression. Notably, macrophages and related factors are considered as the prognostic factors in HCC patients. Since the macrophages can develop an immunosuppressive TME, their therapeutic regulation is of importance for the cancer immunotherapy and this can predict the response to immunotherapeutics in the clinical setting. Overall, macrophages are mainly derived from the circulating monocytes, transforming into macrophages in the TME of HCC. Macrophages can be observed in two phenotypes in TME of HCC including M1 and M2. The polarization of macrophages into M2 phenotype can increase the progression of HCC cells. Notably, M2 polarized macrophages can disrupt the function of other immune cells such as T cells, causing the immune evasion and reducing the efficacy of immunotherapy. There is association between macrophages and biological events. Autophagy status can be changed in the macrophages and due to its dual role, autophagy can induce both M1 and M2 polarization of macrophages. A high number of studies demonstrate that lack of pro-death autophagy in macrophages can increase their M2 polarization. Moreover, glycolysis and EMT are induced in or by macrophages to enhance progression of HCC cells. One of the notable interactions occurs between macrophages and exosomes in HCC. The tumor cells and other kinds of cells have the ability to secrete the exosomes that can finally affect the polarization of macrophages based on the bioactive molecules that they transfer. On the other hand, the macrophages can secrete exosomes for intercellular communication that M2 polarized macrophages secrete exosomes to induce HCC progression. In order to suppress HCC progression, several strategies for regulating macrophages have been deployed. The depletion of macrophages, reduction in the monocyte recruitment, controlling differentiation and suppression of M2 polarization have been applied. Moreover, natural products and nanoparticles have been widely utilized for the regulation of macrophages in the treatment of HCC. Since the natural products suffer from poor bioavailability, the application of nanoparticles for the delivery of natural products and synthetic compounds can improve their selectivity and tumor site release to increase macrophage regulation, promote M1 polarization and enhance macrophage depletion in the treatment of HCC.

TAMs play pivotal roles in HCC progression, existing literature often treats TAMs as a homogeneous group. There's a need for detailed analysis of the diverse functions and phenotypes of TAMs in HCC, especially how different TAM subsets contribute to tumor progression and therapy resistance. Previous studies have noted the influence of TAMs on HCC, but there's less focus on how TAMs interact with other components of the TME, such as fibroblasts, endothelial cells, and other immune cells, and how these interactions contribute to HCC pathology. Although some work has been done on how TAMs affect the efficacy of HCC treatments, there is a gap in understanding the mechanistic pathways through which TAMs mediate resistance to therapies, particularly newer immune therapies. Our study provides a detailed review of the origins, markers, and functions of different TAM subsets in HCC, enhancing understanding of TAM diversity. This helps in pinpointing specific TAM phenotypes that could be targeted to improve therapeutic outcomes. We evaluate the complex interactions between TAMs and other TME components, offering insights into how these interactions promote HCC progression. By mapping out these interactions, your research suggests new therapeutic targets within the TME. Our research explores how different TAM phenotypes contribute to resistance against various HCC treatments. You provide evidence of specific pathways and molecular mechanisms involved, which could be leveraged to develop TAM-targeted therapies that may enhance the efficacy of existing HCC treatments.

One promising area is the targeting of macrophage polarization. Shifting TAM polarization from M2 to M1 could inhibit tumor progression, given M1's anti-tumoral effects and M2's support for tumor growth. Specific molecular targets, such as cytokines crucial in TAM-mediated HCC progression, could be targeted with monoclonal antibodies. Additionally, TAM-related factors could serve as biomarkers for HCC diagnosis or prognosis, helping stratify patients into precise treatment groups for more personalized therapies. Combining TAM modulation with existing therapies like chemotherapy or immunotherapy could enhance their efficacy. Exploring nanoparticle-based drug delivery to target TAMs directly or modulate their activity in the TME might also be effective. The development of these strategies necessitates a robust pathway from preclinical studies in animal models that closely mimic human HCC to clinical trials. Starting with Phase I trials to test safety and dosing, progressing through Phase II and III to evaluate efficacy, and continuously addressing regulatory and ethical considerations are essential steps.

Addressing the limitations of your study on TAMs in HCC is crucial for enhancing the academic rigor of your paper. Acknowledge limitations such as the potential oversimplification in the categorization of TAMs into M1 and M2, which may not capture the full spectrum of TAM phenotypes within the TME. Additionally, discuss the constraints related to model systems used, like *in vitro* studies or animal models, which may not fully replicate human TME dynamics. If applicable, mention limitations related to sample size or diversity that could affect the generalizability of your findings, as well as potential biases in measurement techniques that might influence the outcomes. Proposing directions for future research can significantly extend the impact of your work. Recommend employing more sophisticated models such as patient-derived xenografts or organoids to better mimic the human TME and validate your findings under clinically relevant conditions. Suggest comprehensive TAM profiling using advanced technologies like single-cell RNA sequencing to uncover additional therapeutic targets and understand TAM diversity more deeply. Additionally, encourage the exploration of combination therapies where TAM modulation is integrated with existing HCC treatments to enhance efficacy, and highlight the need for longitudinal studies to assess the long-term effects of such therapies on patient survival and disease progression. Lastly, advocate for studies that examine the influence of genetic and environmental factors on TAM behavior and treatment responses across diverse patient populations. This could facilitate the development of personalized TAM-targeted therapies. By discussing these limitations and future research directions, you not only validate the findings of your current study but also pave the way for future inquiries that can build upon your work, driving forward the field of HCC treatment through innovative approaches to understanding and manipulating TAMs within the TME.

## CRediT authorship contribution statement

**Tianhao Zhang:** Writing – review & editing, Visualization, Project administration, Investigation, Formal analysis, Conceptualization, Writing – original draft, Supervision, Methodology, Funding acquisition, Data curation. **Xi Zhao:** Writing – original draft, Software, Methodology, Funding acquisition, Data curation, Writing – review & editing, Visualization, Resources, Investigation, Formal analysis, Conceptualization. **Tingting Gao:** Writing – original draft, Supervision, Project administration, Investigation, Formal analysis, Conceptualization, Writing – review & editing, Visualization, Software, Methodology, Funding acquisition, Data curation. **Fang Ma:** Writing – review & editing, Visualization, Project administration, Investigation, Formal analysis, Conceptualization, Writing – original draft, Resources, Methodology, Funding acquisition, Data curation.

## Declaration of competing interest

The authors declare that they have no known competing financial interests or personal relationships that could have appeared to influence the work reported in this paper.
