# Supplementary material for: Tumor-associated macrophages in hepatocellular carcinoma: Cellular plasticity and therapy resistance in crosstalk
Source: J Pharm Anal. 2025 Jun 30;16(1):101384. doi: 10.1016/j.jpha.2025.101384 (PMC12856307; doi:10.1016/j.jpha.2025.101384)
Supplement: Multimedia component 1 [file mmc1.docx]

**Table S1:** The versatile function of macrophages in the regulation of hepatocellular carcinoma (HCC) progression.

| **Molecular pathways** | **Remarks** | **Refs.** |
| --- | --- | --- |
| PI3K/Akt | d-lactate and its delivery by nanoparticles can increase M1 polarization of macrophages to remodel TME and enhance potential of anti-CD47 antibody | [1] |
| SLAMF7/CCL2 | SLAMF7 impairs MAPK/ATF2/CCl2 axis to increase immunotherapy sensitivity | [2] |
| - | Lenvatinib promotes NAD^+^ synthesis in reducing M2 polarization | [3] |
| NF-κB | The inflammatory macrophages require NF-κB to diminish the efficacy of DOT1L suppression | [4] |
| PIM2 | Pro-inflammatory macrophages stimulate PIM2 expression to disrupt immunotherapy | [5] |
| - | PD-L1+ host macrophages demonstrate an HLA-DRhighCD86high glycolytic phenotype and their polarization is regulated by glycolysis | [6] |
| FOXO1 | FOXO1 derived from HCC cells can impair IL-6 secretion from macrophages to reduce tumorigenesis | [7] |
| MYC | Upregulation of MYC causes immune evasion through increasing levels of pro-inflammatory macrophages | [8] |
| IL-17 | The presence of IL-17 signaling in steatotic hepatocytes and macrophages can enhance HCC development | [9] |
| - | Increase in the lung metastasis of HCC cells is mediated by the alveolar macrophages producing leukotriene B_4_ | [10] |
| IL-37 | IL-37 impairs the M2 polarization of macrophages to reduce HCC proliferation | [11] |
| - | The TAMs have shown potential in impairing the function of MAIT cell in the metastatic region of HCC cells | [12] |
| OIT3 | Upregulation of OIT3 can upregulate PD-L1 expression through NF-κB upregulation | [13] |
| PLA2G7 | PLA2G7 downregulation disrupt the immunosuppressive function of macrophages | [14] |
| hsa_circ_0003410 | hsa_circ_0003410 enhances M2 polarization of macrophages through CCL5 upregulation | [15] |
| miR-660-5p | Exosomes derived from M2 macrophages enriched with miR-660-5p can increase HCC progression via KLF3 inhibition | [16] |
| - | The aggressive feature of HCC cells can be achieved through poor infiltration of CD86+ TAMs and increased number of CD206+ TAMs | [17] |
| - | The TAMs and Treg cells can be recruited by cancer-associated fibroblasts in HCC resistance to sorafenib | [18] |
| - | The efflux of cholesterol can provide immunosuppressive macrophages | [19] |
| - | Autophagy induction by macrophages triggers oxaliplatin resistance | [20] |
| STAT3 | TAMs use STAT3 axis to generate IL-6 in HCC progression | [21] |
| IL-6 | The IL-6 depletion in monocytes and macrophages can impair the development of HCC in mice | [22] |
| CCL7 | Secretion of CCL2 by macrophages in cancer metastasis | [23] |
| YAP | YAP mediates IL-6 release by HCC cells to recruit TAMs | [24] |
| APOC1 | APOC1 downregulation increases M1 polarization of macrophages through ferroptosis and promote the function of anti-PD-1 therapy | [25] |
| - | Onco-fetal reprogramming of endothelial cells enhances the immunosuppressive activity of macrophages | [26] |
| PD-L1 | M1 polarized macrophages increase PD-L1 levels via IL-1β | [27] |
| CXCR2 | TAMs induce CXCR2 axis to mediate sorafenib resistance | [28] |
| PAI-1 | Cancer-associated fibroblasts stimulate M2 polarization of macrophages with upregulation of PAI-1 | [29] |
| - | TREM2+ macrophages can impair the infiltration of CD8+ T cells | [30] |
| IGF-1 | Sorafenib impairs the secretion of IGF-1 to reduce macrophage-mediated proliferation of hepatoma cells | [31] |
| PPARγ | Genipin-activating PPARγ prevents the relapse of HCC through suppressing CCR2-induced macrophage infiltration | [32] |
| - | Cinobufacini injection disrupts M2 polarization of macrophages mediated by IL-4 | [33] |
| B3GALNT2 | B3GALNT2 upregulation enhances the recruitment of macrophages through decreasing acetoacetate secretion and enhancing MIF function | [34] |
| miR-452-5p | Exosomal miR-452-5p controls TIMP3 to enhance M2 polarization of macrophages | [35] |
| FAK | FAK mediates Treg cells to induce M2 polarization of macrophages | [36] |
| SIRT1 | SIRT1 promotes M1 polarization of macrophages through NF-κB upregulation to reduce metastasis | [37] |
| RIG-I | RIG-I enhances M1 polarization of macrophages via upregulating MAVS and TRAF2 to mediate cell death | [38] |
| TOP2A | CD168+ macrophages can accelerate tumorigenesis and stemness through promoting dissociation of β-catenin from YAP mediated by TOP2A | [39] |
| - | Astragalus polysacharin impairs the M2 polarization of macrophages | [40] |
| CXCR4 | Monocytes and macrophages induce ERK axis to enhance CXCR4 levels | [41] |
| - | Estrogen impairs the induction of TAMs to limit HCC proliferation | [42] |
| CXCL16/CXCR2 | Induction of CXCL16/CXCR2 axis by macrophages can increase progression and radioresistance | [43] |
| p38 kinase/Creb1/Klf4 | Regorafenib suppresses p38 kinase/Creb1/Klf4 in TAMs to enhance anti-cancer immunity | [44] |
| JUN | Fibronectin 1 secreted by TAMs and fibroblasts upregulate JUN to elevate metastasis and invasion | [45] |
| OXCT1 | OXCT1 induces ketone metabolism to reprogram TAMs in reducing anti-cancer immunity through CD8+ T cell exhaustion | [46] |
| **Abbreviations**: PI3K/Akt: Phosphoinositide 3-kinase/Protein Kinase B, SLAMF7: Signaling Lymphocytic Activation Molecule Family member 7, CCL2: Chemokine (C-C motif) ligand 2, MAPK: Mitogen-Activated Protein Kinase, ATF2: Activating Transcription Factor 2, NF-κB: Nuclear Factor Kappa B, DOT1L: Disruptor of Telomeric Silencing 1-Like, PIM2: Proviral Integration site for Moloney murine leukemia virus 2, PD-L1: Programmed Death-Ligand 1, HLA-DR: Human Leukocyte Antigen - DR isotype, CD86: Cluster of Differentiation 86, FOXO1: Forkhead Box O1, HCC: Hepatocellular Carcinoma, IL-6: Interleukin 6, MYC: MYC Proto-Oncogene, IL-17: Interleukin 17, IL-37: Interleukin 37, TAMs: Tumor-Associated Macrophages, MAIT: Mucosal-Associated Invariant T cells, OIT3: Oncoprotein-Induced Transcript 3, PLA2G7: Phospholipase A2 Group VII, hsa_circ_0003410: Circular RNA 0003410, CCL5: Chemokine (C-C motif) ligand 5, miR-660-5p: MicroRNA-660-5p, KLF3: Kruppel-Like Factor 3, CD206: Cluster of Differentiation 206, Treg: Regulatory T cells, STAT3: Signal Transducer and Activator of Transcription 3, CCL7: Chemokine (C-C motif) ligand 7, YAP: Yes-Associated Protein, APOC1: Apolipoprotein C1, PD-1: Programmed Cell Death Protein 1, CXCR2: C-X-C Motif Chemokine Receptor 2, PAI-1: Plasminogen Activator Inhibitor-1, TREM2: Triggering Receptor Expressed on Myeloid cells 2, IGF-1: Insulin-Like Growth Factor 1, PPARγ: Peroxisome Proliferator-Activated Receptor Gamma, CCR2: C-C Chemokine Receptor Type 2, B3GALNT2: Beta-1,3-N-Acetylgalactosaminyltransferase 2, MIF: Macrophage Migration Inhibitory Factor, miR-452-5p: MicroRNA-452-5p, TIMP3: Tissue Inhibitor of Metalloproteinases 3, FAK: Focal Adhesion Kinase, SIRT1: Sirtuin 1, RIG-I: Retinoic Acid-Inducible Gene I, MAVS: Mitochondrial Antiviral-Signaling Protein, TRAF2: TNF Receptor-Associated Factor 2, TOP2A: Topoisomerase II Alpha, CXCR4: C-X-C Motif Chemokine Receptor 4, ERK: Extracellular Signal-Regulated Kinase, CXCL16: C-X-C Motif Chemokine Ligand 16, p38 kinase: p38 Mitogen-Activated Protein Kinase, Creb1: cAMP Responsive Element Binding Protein 1, Klf4: Kruppel-Like Factor 4, JUN: Jun Proto-Oncogene, OXCT1: 3-Oxoacid CoA-Transferase 1. | | |

**Table S2:** An overview of nanoparticle application in TAM regulation in HCC therapy.

| **Nanoparticle** | **Remark** | **Reference** |
| --- | --- | --- |
| Liposomes | Cancer-associated fibroblasts (CAFs) deteriorate the tumor microenvironment and hinder drug delivery. Reversing the EMT can help combat hepatocellular carcinoma. A CFH peptide-decorated liposomal oxymatrine (CFH/OM-L) has been found to inactivate CAFs through EMT, reducing tumor metastasis risk and reprogramming the tumor microenvironment. This strategy offers a moderate way to remold the tumor microenvironment without depleting CAFs, providing a powerful tool for combinational hepatocellular carcinoma therapy. | [47] |
| Nanohydrogel | A new nanoplatform, HMSN@Sor/aP@Gel, has been developed to prevent postoperative recurrence and metastasis of HCC. The platform combines a hemostatic hydrogel with functionalized hollow mesoporous silica nanoparticles loaded with sorafenib and anti-PD-L1. The antitumor mechanism involves dual inhibition of Ras/Raf/MEK/ERK and PI3K/AKT pathways, complemented by PD-L1 blockade. Animal studies show that this targeted immunotherapy significantly impedes tumor growth and establishes immune memory, indicating potential for clinical translation. | [48] |
| NanoMnSor | The study presents NanoMnSor, a nanoparticle drug carrier that efficiently codeslives oxygen-generating MnO_2_ and sorafenib into advanced HCC. MnO_2_ alleviates hypoxia, enhances T1-weighted magnetic resonance imaging, and improves overall survival in a mouse orthotopic HCC model. NanoMnSor also reprograms the immunosuppressive TME, reducing hypoxia-induced tumor infiltration and increasing CD^8+^ cytotoxic T cells, thus enhancing the efficacy of anti-PD-1 antibody and whole-cell cancer vaccine immunotherapies. | [49] |
| Mesoporous Fe_3_O_4_ nanoparticles | An efficient immunovascular crosstalk modulation strategy could help combat HCC by reversing immunosuppression and vessel normalization, especially by reprogramming TAMs. This study uses tyrosine kinase inhibitor lenvatinib in mesoporous Fe3O4 nanoparticles and bovine serum albumin to create a metallodrug. | [50] |
| PLGA nanostructures | Sorafenib, a multikinase inhibitor, has been used to treat highly vascular HCC but has been linked to recurrence. Researchers have developed CXCR4-targeted nanoparticles (NPs) to deliver sorafenib into HCC and sensitize it to treatment. The NPs effectively deliver sorafenib, achieving cytotoxicity and anti-angiogenic effects in vitro and in vivo. AMD3100, attached to the NPs, blocks CXCR4/SDF1α, reducing tumor infiltration and enhancing anti-angiogenic effects. | [51] |
| Chromium nanostructures | A photosensitive, dual-targeting nanoparticle system (M.RGD@Cr-CTS-siYTHDF1 NPs) was developed to target TAMs and cancer cells. The system includes DSPE-modified RGD peptides targeting integrin receptors on tumor cells and CD206 receptors on macrophages, with chitosan adsorbing m6A reading protein YTHDF1 siRNA and chromium nanoparticles. Experiments with tumor-bearing mice showed that the system effectively killed tumor cells, disrupted the tumor microenvironment, and delivered siYTHDF1 to TAMs, leading to an anti-tumor phenotype. | [52] |
| Lipid nanocarriers | This study presents a CXCR4-targeted lipid-based nanoparticle formulation for treating HCC. The nanoparticles deliver VEGF siRNA as an antiangiogenic substance, with AMD3100 added as a CXCR4 antagonist. AMD-NPs efficiently deliver VEGF siRNAs into HCC, downregulate VEGF expression, and prevent tumor infiltration. This approach overcomes tumor evasion of antiangiogenic therapy, leading to delayed tumor progression in HCC. | [53] |
| **Abbreviations:** CAFs: Cancer-Associated Fibroblasts, EMT: Epithelial-Mesenchymal Transition, CFH/OM-L: CFH peptide-decorated liposomal oxymatrine, HCC: Hepatocellular Carcinoma, HMSN@Sor/aP@Gel: Hemostatic hydrogel with functionalized hollow mesoporous silica nanoparticles loaded with sorafenib and anti-PD-L1, Ras/Raf/MEK/ERK: Ras/Raf/Mitogen-Activated Protein Kinase/Extracellular Signal-Regulated Kinase, PI3K/AKT: Phosphoinositide 3-kinase/Protein Kinase B, PD-L1: Programmed Death-Ligand 1, NanoMnSor: Nanoparticle drug carrier with MnO2 and sorafenib, TME: Tumor Microenvironment, CD8+: Cluster of Differentiation 8 positive, Fe3O4: Iron (II, III) Oxide, PLGA: Poly(lactic-co-glycolic acid), CXCR4: C-X-C Motif Chemokine Receptor 4, SDF1α: Stromal Cell-Derived Factor 1 alpha, AMD3100: Plerixafor, M.RGD@Cr-CTS-siYTHDF1 NPs: Photosensitive, dual-targeting nanoparticle system with RGD peptides, chitosan, and chromium nanoparticles, DSPE: 1,2-Distearoyl-sn-glycero-3-phosphoethanolamine, RGD: Arginylglycylaspartic acid, CD206: Cluster of Differentiation 206, siYTHDF1: Small interfering RNA targeting YTH N6-Methyladenosine RNA Binding Protein 1, VEGF: Vascular Endothelial Growth Factor, siRNA: Small interfering RNA, AMD-NPs: AMD3100-conjugated nanoparticles. | | |

**References:**

[1] S. Han, X. Bao, Y. Zou, et al., d-lactate modulates M2 tumor-associated macrophages and remodels immunosuppressive tumor microenvironment for hepatocellular carcinoma, Sci Adv 9 (2023) eadg2697 <https://doi.org/10.1126/sciadv.adg2697>.

[2] J. Weng, Z. Wang, Z. Hu, et al., Repolarization of Immunosuppressive Macrophages by Targeting SLAMF7-Regulated CCL2 Signaling Sensitizes Hepatocellular Carcinoma to Immunotherapy, Cancer research 84 (2024) 1817-1833 <https://doi.org/10.1158/0008-5472.can-23-3106>.

[3] Q. Sun, M. Shen, S. Zhu, et al., Targeting NAD(+) metabolism of hepatocellular carcinoma cells by lenvatinib promotes M2 macrophages reverse polarization, suppressing the HCC progression, Hepatology international 17 (2023) 1444-1460 <https://doi.org/10.1007/s12072-023-10544-7>.

[4] Y.B. Yang, C.Y. Wu, X.Y. Wang, et al., Targeting inflammatory macrophages rebuilds therapeutic efficacy of DOT1L inhibition in hepatocellular carcinoma, Molecular therapy : the journal of the American Society of Gene Therapy 31 (2023) 105-118 <https://doi.org/10.1016/j.ymthe.2022.09.019>.

[5] J.C. Wang, D.P. Chen, S.X. Lu, et al., PIM2 Expression Induced by Proinflammatory Macrophages Suppresses Immunotherapy Efficacy in Hepatocellular Carcinoma, Cancer research 82 (2022) 3307-3320 <https://doi.org/10.1158/0008-5472.can-21-3899>.

[6] L.G. Lu, Z.L. Zhou, X.Y. Wang, et al., PD-L1 blockade liberates intrinsic antitumourigenic properties of glycolytic macrophages in hepatocellular carcinoma, Gut 71 (2022) 2551-2560 <https://doi.org/10.1136/gutjnl-2021-326350>.

[7] X. Cui, H. Zhao, S. Wei, et al., Hepatocellular carcinoma-derived FOXO1 inhibits tumor progression by suppressing IL-6 secretion from macrophages, Neoplasia (New York, N.Y.) 40 (2023) 100900 <https://doi.org/10.1016/j.neo.2023.100900>.

[8] R. Dhanasekaran, A.S. Hansen, J. Park, et al., MYC Overexpression Drives Immune Evasion in Hepatocellular Carcinoma That Is Reversible through Restoration of Proinflammatory Macrophages, Cancer research 83 (2023) 626-640 <https://doi.org/10.1158/0008-5472.can-22-0232>.

[9] H.Y. Ma, G. Yamamoto, J. Xu, et al., IL-17 signaling in steatotic hepatocytes and macrophages promotes hepatocellular carcinoma in alcohol-related liver disease, Journal of hepatology 72 (2020) 946-959 <https://doi.org/10.1016/j.jhep.2019.12.016>.

[10] T. Nosaka, T. Baba, Y. Tanabe, et al., Alveolar Macrophages Drive Hepatocellular Carcinoma Lung Metastasis by Generating Leukotriene B(4), Journal of immunology (Baltimore, Md. : 1950) 200 (2018) 1839-1852 <https://doi.org/10.4049/jimmunol.1700544>.

[11] Z. Zhang, J. Zhang, P. He, et al., Interleukin-37 suppresses hepatocellular carcinoma growth through inhibiting M2 polarization of tumor-associated macrophages, Molecular immunology 122 (2020) 13-20 <https://doi.org/10.1016/j.molimm.2020.03.012>.

[12] B. Ruf, M. Bruhns, S. Babaei, et al., Tumor-associated macrophages trigger MAIT cell dysfunction at the HCC invasive margin, Cell 186 (2023) 3686-3705.e3632 <https://doi.org/10.1016/j.cell.2023.07.026>.

[13] J. Wen, S. Yang, G. Yan, et al., Increased OIT3 in macrophages promotes PD-L1 expression and hepatocellular carcinogenesis via NF-κB signaling, Experimental cell research 428 (2023) 113651 <https://doi.org/10.1016/j.yexcr.2023.113651>.

[14] F. Zhang, W. Liu, F. Meng, et al., Inhibiting PLA2G7 reverses the immunosuppressive function of intratumoral macrophages and augments immunotherapy response in hepatocellular carcinoma, Journal for immunotherapy of cancer 12 (2024) <https://doi.org/10.1136/jitc-2023-008094>.

[15] P. Cao, B. Ma, D. Sun, et al., hsa_circ_0003410 promotes hepatocellular carcinoma progression by increasing the ratio of M2/M1 macrophages through the miR-139-3p/CCL5 axis, Cancer science 113 (2022) 634-647 <https://doi.org/10.1111/cas.15238>.

[16] B. Tian, L. Zhou, J. Wang, et al., miR-660-5p-loaded M2 macrophages-derived exosomes augment hepatocellular carcinoma development through regulating KLF3, International immunopharmacology 101 (2021) 108157 <https://doi.org/10.1016/j.intimp.2021.108157>.

[17] P. Dong, L. Ma, L. Liu, et al., CD86⁺/CD206⁺, Diametrically Polarized Tumor-Associated Macrophages, Predict Hepatocellular Carcinoma Patient Prognosis, International journal of molecular sciences 17 (2016) 320 <https://doi.org/10.3390/ijms17030320>.

[18] S.L. Zhou, Z.J. Zhou, Z.Q. Hu, et al., Tumor-Associated Neutrophils Recruit Macrophages and T-Regulatory Cells to Promote Progression of Hepatocellular Carcinoma and Resistance to Sorafenib, Gastroenterology 150 (2016) 1646-1658.e1617 <https://doi.org/10.1053/j.gastro.2016.02.040>.

[19] Z. Li, Y. Wang, R. Xing, et al., Cholesterol Efflux Drives the Generation of Immunosuppressive Macrophages to Promote the Progression of Human Hepatocellular Carcinoma, Cancer immunology research 11 (2023) 1400-1413 <https://doi.org/10.1158/2326-6066.cir-22-0907>.

[20] X.T. Fu, K. Song, J. Zhou, et al., Tumor-associated macrophages modulate resistance to oxaliplatin via inducing autophagy in hepatocellular carcinoma, Cancer cell international 19 (2019) 71 <https://doi.org/10.1186/s12935-019-0771-8>.

[21] S. Wan, E. Zhao, I. Kryczek, et al., Tumor-associated macrophages produce interleukin 6 and signal via STAT3 to promote expansion of human hepatocellular carcinoma stem cells, Gastroenterology 147 (2014) 1393-1404 <https://doi.org/10.1053/j.gastro.2014.08.039>.

[22] L. Kong, Y. Zhou, H. Bu, et al., Deletion of interleukin-6 in monocytes/macrophages suppresses the initiation of hepatocellular carcinoma in mice, Journal of experimental & clinical cancer research : CR 35 (2016) 131 <https://doi.org/10.1186/s13046-016-0412-1>.

[23] S. Huang, L. He, Y. Zhao, et al., TREM1(+) tumor-associated macrophages secrete CCL7 to promote hepatocellular carcinoma metastasis, Journal of cancer research and clinical oncology 150 (2024) 320 <https://doi.org/10.1007/s00432-024-05831-1>.

[24] T.Y. Zhou, Y.L. Zhou, M.J. Qian, et al., Interleukin-6 induced by YAP in hepatocellular carcinoma cells recruits tumor-associated macrophages, Journal of pharmacological sciences 138 (2018) 89-95 <https://doi.org/10.1016/j.jphs.2018.07.013>.

[25] X. Hao, Z. Zheng, H. Liu, et al., Inhibition of APOC1 promotes the transformation of M2 into M1 macrophages via the ferroptosis pathway and enhances anti-PD1 immunotherapy in hepatocellular carcinoma based on single-cell RNA sequencing, Redox biology 56 (2022) 102463 <https://doi.org/10.1016/j.redox.2022.102463>.

[26] A. Sharma, J.J.W. Seow, C.A. Dutertre, et al., Onco-fetal Reprogramming of Endothelial Cells Drives Immunosuppressive Macrophages in Hepatocellular Carcinoma, Cell 183 (2020) 377-394.e321 <https://doi.org/10.1016/j.cell.2020.08.040>.

[27] Z. Zong, J. Zou, R. Mao, et al., M1 Macrophages Induce PD-L1 Expression in Hepatocellular Carcinoma Cells Through IL-1β Signaling, Frontiers in immunology 10 (2019) 1643 <https://doi.org/10.3389/fimmu.2019.01643>.

[28] H.C. Wang, L.Y. Haung, C.J. Wang, et al., Tumor-associated macrophages promote resistance of hepatocellular carcinoma cells against sorafenib by activating CXCR2 signaling, Journal of biomedical science 29 (2022) 99 <https://doi.org/10.1186/s12929-022-00881-4>.

[29] S. Chen, Y. Morine, K. Tokuda, et al., Cancer‑associated fibroblast‑induced M2‑polarized macrophages promote hepatocellular carcinoma progression via the plasminogen activator inhibitor‑1 pathway, International journal of oncology 59 (2021) <https://doi.org/10.3892/ijo.2021.5239>.

[30] J. Tan, W. Fan, T. Liu, et al., TREM2(+) macrophages suppress CD8(+) T-cell infiltration after transarterial chemoembolisation in hepatocellular carcinoma, Journal of hepatology 79 (2023) 126-140 <https://doi.org/10.1016/j.jhep.2023.02.032>.

[31] M.F. Sprinzl, A. Puschnik, A.M. Schlitter, et al., Sorafenib inhibits macrophage-induced growth of hepatoma cells by interference with insulin-like growth factor-1 secretion, Journal of hepatology 62 (2015) 863-870 <https://doi.org/10.1016/j.jhep.2014.11.011>.

[32] J. Wu, Y.T. Chan, Y. Lu, et al., Genipin-activating PPARγ impedes CCR2-mediated macrophage infiltration into postoperative liver to suppress recurrence of hepatocellular carcinoma, International journal of biological sciences 19 (2023) 5257-5274 <https://doi.org/10.7150/ijbs.87327>.

[33] M. Wang, Y. Li, S. Li, et al., Cinobufacini injection delays hepatocellular carcinoma progression by regulating lipid metabolism via SREBP1 signaling pathway and affecting macrophage polarization, Journal of ethnopharmacology 321 (2024) 117472 <https://doi.org/10.1016/j.jep.2023.117472>.

[34] T. Yang, Y. Wang, W. Dai, et al., Increased B3GALNT2 in hepatocellular carcinoma promotes macrophage recruitment via reducing acetoacetate secretion and elevating MIF activity, J Hematol Oncol 11 (2018) 50 <https://doi.org/10.1186/s13045-018-0595-3>.

[35] H. Zongqiang, C. Jiapeng, Z. Yingpeng, et al., Exosomal miR-452-5p Induce M2 Macrophage Polarization to Accelerate Hepatocellular Carcinoma Progression by Targeting TIMP3, Journal of immunology research 2022 (2022) 1032106 <https://doi.org/10.1155/2022/1032106>.

[36] F. Chen, M. Gong, D. Weng, et al., Phellinus linteus activates Treg cells via FAK to promote M2 macrophage polarization in hepatocellular carcinoma, Cancer immunology, immunotherapy : CII 73 (2024) 18 <https://doi.org/10.1007/s00262-023-03592-3>.

[37] B. Zhou, Y. Yang, C. Li, SIRT1 inhibits hepatocellular carcinoma metastasis by promoting M1 macrophage polarization via NF-κB pathway, OncoTargets and therapy 12 (2019) 2519-2529 <https://doi.org/10.2147/ott.s195234>.

[38] B. Zhou, C. Li, Y. Yang, et al., RIG-I Promotes Cell Death in Hepatocellular Carcinoma by Inducing M1 Polarization of Perineal Macrophages Through the RIG-I/MAVS/NF-κB Pathway, OncoTargets and therapy 13 (2020) 8783-8794 <https://doi.org/10.2147/ott.s258450>.

[39] H.C. Zhao, C.Z. Chen, Y.Z. Tian, et al., CD168(+) macrophages promote hepatocellular carcinoma tumor stemness and progression through TOP2A/β-catenin/YAP1 axis, iScience 26 (2023) 106862 <https://doi.org/10.1016/j.isci.2023.106862>.

[40] C. Li, X.Y. Pan, M. Ma, et al., Astragalus polysacharin inhibits hepatocellular carcinoma-like phenotypes in a murine HCC model through repression of M2 polarization of tumour-associated macrophages, Pharmaceutical biology 59 (2021) 1533-1539 <https://doi.org/10.1080/13880209.2021.1991384>.

[41] Y.M. Meng, J. Liang, C. Wu, et al., Monocytes/Macrophages promote vascular CXCR4 expression via the ERK pathway in hepatocellular carcinoma, Oncoimmunology 7 (2018) e1408745 <https://doi.org/10.1080/2162402x.2017.1408745>.

[42] W. Yang, Y. Lu, Y. Xu, et al., Estrogen represses hepatocellular carcinoma (HCC) growth via inhibiting alternative activation of tumor-associated macrophages (TAMs), The Journal of biological chemistry 287 (2012) 40140-40149 <https://doi.org/10.1074/jbc.M112.348763>.

[43] H.L. Lee, Y.C. Tsai, N.W. Pikatan, et al., Tumor-Associated Macrophages Affect the Tumor Microenvironment and Radioresistance via the Upregulation of CXCL6/CXCR2 in Hepatocellular Carcinoma, Biomedicines 11 (2023) <https://doi.org/10.3390/biomedicines11072081>.

[44] D.L. Ou, C.W. Chen, C.L. Hsu, et al., Regorafenib enhances antitumor immunity via inhibition of p38 kinase/Creb1/Klf4 axis in tumor-associated macrophages, Journal for immunotherapy of cancer 9 (2021) <https://doi.org/10.1136/jitc-2020-001657>.

[45] L. Zhang, C. Zhang, Z. Xing, et al., Fibronectin 1 derived from tumor-associated macrophages and fibroblasts promotes metastasis through the JUN pathway in hepatocellular carcinoma, International immunopharmacology 113 (2022) 109420 <https://doi.org/10.1016/j.intimp.2022.109420>.

[46] C.X. Zhu, K. Yan, L. Chen, et al., Targeting OXCT1-mediated ketone metabolism reprograms macrophages to promote antitumor immunity via CD8(+) T cells in hepatocellular carcinoma, Journal of hepatology (2024) <https://doi.org/10.1016/j.jhep.2024.05.007>.

[47] J. Guo, H. Zeng, X. Shi, et al., A CFH peptide-decorated liposomal oxymatrine inactivates cancer-associated fibroblasts of hepatocellular carcinoma through epithelial-mesenchymal transition reversion, Journal of nanobiotechnology 20 (2022) 114 <https://doi.org/10.1186/s12951-022-01311-1>.

[48] J. Ke, Y. Liu, F. Liu, et al., In-situ-formed immunotherapeutic and hemostatic dual drug-loaded nanohydrogel for preventing postoperative recurrence of hepatocellular carcinoma, Journal of controlled release : official journal of the Controlled Release Society 372 (2024) 141-154 <https://doi.org/10.1016/j.jconrel.2024.06.030>.

[49] C.C. Chang, T.K. Dinh, Y.A. Lee, et al., Nanoparticle Delivery of MnO(2) and Antiangiogenic Therapy to Overcome Hypoxia-Driven Tumor Escape and Suppress Hepatocellular Carcinoma, ACS applied materials & interfaces 12 (2020) 44407-44419 <https://doi.org/10.1021/acsami.0c08473>.

[50] Y. Luo, J. Wang, L. Xu, et al., A theranostic metallodrug modulates immunovascular crosstalk to combat immunosuppressive liver cancer, Acta biomaterialia 154 (2022) 478-496 <https://doi.org/10.1016/j.actbio.2022.10.032>.

[51] D.Y. Gao, T. Lin Ts, Y.C. Sung, et al., CXCR4-targeted lipid-coated PLGA nanoparticles deliver sorafenib and overcome acquired drug resistance in liver cancer, Biomaterials 67 (2015) 194-203 <https://doi.org/10.1016/j.biomaterials.2015.07.035>.

[52] S. Chen, Y. He, X. Huang, et al., Photosensitive and dual-targeted chromium nanoparticle delivering small interfering RNA YTHDF1 for molecular-targeted immunotherapy in liver cancer, Journal of nanobiotechnology 22 (2024) 348 <https://doi.org/10.1186/s12951-024-02612-3>.

[53] J.Y. Liu, T. Chiang, C.H. Liu, et al., Delivery of siRNA Using CXCR4-targeted Nanoparticles Modulates Tumor Microenvironment and Achieves a Potent Antitumor Response in Liver Cancer, Molecular therapy : the journal of the American Society of Gene Therapy 23 (2015) 1772-1782 <https://doi.org/10.1038/mt.2015.147>.
